# An Application for Spatial Frailty Models: An Exploration with Data on Fungal Sepsis in Neonates

**DOI:** 10.3390/diseases13030083

**Published:** 2025-03-14

**Authors:** Palaniyandi Paramasivam, Nagaraj Jaganathasamy, Srinivasan Ramalingam, Vasantha Mahalingam, Selvam Nagarajan, Fayaz Ahamed Shaik, Sundarakumar Karuppasamy, Adhin Bhaskar, Padmanaban Srinivasan, Tamizhselvan Manoharan, Adalarasan Natesan, Ponnuraja Chinnaiyan

**Affiliations:** 1 Department of Statistics, ICMR—National Institute for Research in Tuberculosis, Chennai 600 031, Tamil Nadu, India; palani.stats@gmail.com (P.P.); vasanthabio@gmail.com (V.M.); selvam.nagarajan@gmail.com (S.N.); ahamedsfayaz@gmail.com (F.A.S.); sundarakumar6118@gmail.com (S.K.); adhinb6001@gmail.com (A.B.); padmanaban17@yahoo.com (P.S.); elantamizh@gmail.com (T.M.); 2 University of Madras, Chennai 600 005, Tamil Nadu, India; 3 Department of Biostatistics, ICMR—National Institute of Immunohaemotology (NIIH), Mumbai 400 012, Maharashtra, India; nagarajicmr@gmail.com; 4 Department of Epidemiology and Biostatistics, ICMR—National Institute of Epidemiology, Chennai 600 077, Tamil Nadu, India; ramalingamsrini6@gmail.com; 5 Department of Pediatrics, Government Kilpauk Medical College, Chennai 600 010, Tamil Nadu, India; nadaladav@gmail.com

**Keywords:** spatial, Bayesian, frailty, hazard, activated thromboplastin, neonatal sepsis

## Abstract

Background: Globally, neonatal fungal sepsis (NFS) is a leading cause of neonatal mortality, particularly among vulnerable populations in neonatal intensive care units (NICU). The use of spatial frailty models with a Bayesian approach to identify hotspots and risk factors for neonatal deaths due to fungal sepsis has not been explored before. Methods: A cohort of 80 neonates admitted to the NICU at a Government Hospital in Tamil Nadu, India and diagnosed with fungal sepsis through blood cultures between 2018–2020 was considered for this study. Bayesian spatial frailty models using parametric distributions, such as Log-logistic, Log-normal, and Weibull proportional hazard (PH) models, were employed to identify associated risk factors for NFS deaths and hotspot areas using the R version 4.1.3 software and QGIS version 3.26 (Quantum Geographic Information System). Results: The spatial parametric frailty models were found to be good models for analyzing NFS data. Abnormal levels of activated thromboplastin carried a significantly higher risk of death in neonates across all PH models (Log-logistic, Hazard Ratio (HR), 95% Credible Interval (CI): 22.12, (5.40, 208.08); Log-normal: 20.87, (5.29, 123.23); Weibull: 18.49, (5.60, 93.41). The presence of hemorrhage also carried a risk of death for the Log-normal (1.65, (1.05, 2.75)) and Weibull models (1.75, (1.07, 3.12)). Villivakkam, Tiruvallur, and Poonamallee blocks were identified as high-risk areas. Conclusions: The spatial parametric frailty models proved their effectiveness in identifying these risk factors and quantifying their association with mortality. The findings from this study underline the importance of the early detection and management of risk factors to improve survival outcomes in neonates with fungal sepsis.

## 1. Introduction

Neonatal sepsis (NS) is described as a bloodstream infection that occurs within 28 days after birth and is caused by bacterial, viral, or fungal pathogens [1]. NS was responsible for 15% of newborn deaths worldwide in 2016 [2]. A recent systematic review and meta-analysis of population-based research from various parts of the world estimated an incidence of 2824 newborn sepsis cases per 100,000 live births and a death rate of 17.6% [3]. An estimated 2.3 million newborn deaths occurred worldwide in 2022 [4]. The National Family Health Survey (NFHS-5) covering the period from 2019 to 2021 reported that the neonatal mortality rate (NNMR) in Tamil Nadu was 12.7 per 1000 live births, while the NNMR in India was 24.9 per 1000 live births [5].

Preterm newborns and those with extremely low birth weights were more susceptible to morbidity and death from NS [1,6,7]. More than 1 million newborn infants die every year before completing their first four weeks of life, resulting in the highest burden of newborn deaths for any country in the world [8]. It is especially important to rule out fungal sepsis when a baby is severely ill, even if blood cultures are negative. This syndrome is common in neonatal intensive care units (NICUs), especially when invasive procedures have been performed and prolonged empirical antibiotic treatments have been administered. Neonatal fatalities are caused by sepsis, highlighting the critical need for enhanced infection control and medical treatment for newborns.

A study from Chennai, India concluded that careful monitoring of the coagulation profile, including Prothrombin Time (PT), activated Partial Thromboplastin Clotting Time (aPTT), and Intracranial hemorrhage (ICH), had a substantial impact on non-survival in fungal sepsis neonates when employing Cox regression analysis. The prevalence of fungal infection in infants has varied substantially between medical facilities due to differences in modifiable risk factors [9]. Time-to-event data collected across several geographical areas were frequently divided into strata, or clusters, such as regions or healthcare facilities [10,11]. The occurrence of NFS is Late-Onset Sepsis (LOS).

Frailty models employ random effects to reflect unobserved heterogeneity in survival analysis, helping to explain variability in survival times that cannot be explained by observable factors. These models have been especially useful in situations involving clustered or grouped data, such as patients from several geographical areas [12]. Incorporating geographical information into the survival model will be helpful, if survival times differ between locations. Numerous studies have described the importance of geographic location information in survival prediction and spatial survival frailty models [10,11,13,14,15,16]. An earlier investigation has focused on the patterns and geographic distribution of newborn sepsis in Uganda from 2016 to 2020, with the aim of informing measures to reduce the prevalence of sepsis-related fatalities and neonatal sepsis [17]. Kibret et al., (2022) have also explored the spatial variations and contributing factors for neonatal mortality rates in Ethiopia using Bayesian spatial logistic regression model [18].

The use of spatial survival analysis for figuring out diagnostic delays in high-incidence locations has been demonstrated through an analysis of tuberculosis (TB) diagnosis delays [19]. Numerous studies applied Bayesian spatial survival models to various disease datasets [10,11,14,15,19,20,21,22,23]. To our knowledge, a spatial frailty model using a Bayesian approach to identify hotspot areas and risk factors for neonatal deaths due to fungal sepsis has not yet been studied.

Compared to conventional statistical approaches, the Bayesian spatial method assists in reducing bias and variance more effectively [24]. Advancements in computer technology, such as Geographic Information Systems and Markov chain Monte Carlo (MCMC) methods, have made it easier to implement Bayesian approaches to frailty models, which have been helpful in explaining spatial clustering [11,25]. The Bayesian spatial survival model provides robust estimates when survival data are spatially correlated, complex, and when incorporating prior knowledge and quantifying uncertainty is necessary [13,23].

This was a unique opportunity to use data on newborns with fungal sepsis. Against this background, our research hypothesis aims to identify factors influencing neonatal deaths due to fungal sepsis and to pinpoint hotspot areas associated with these deaths using Bayesian spatial frailty models.

## 2. Materials and Methods

### 2.1. Data Source

Secondary data on 80 neonates who were admitted to the NICU at the Government Kilpauk Medical College and Hospital in Tamil Nadu, India between January 2018 and December 2020 and diagnosed with fungal sepsis using blood cultures [9] were considered for this study. The occurrences of these NFS cases were LOS. Of the 80 neonates, 50 (62.5%) had died, and the remaining 30 (37.5%) were discharged. The variables extracted for this study included the addresses of the neonates, the weeks of gestational age (GA_weeks), the birth weight of the neonates (B_Weight), the presence of hemorrhages, platelet counts (in 10^3^ µL), levels of activated thromboplastin (PT_APTT), the number of hours spent in the NICU (time), and the outcome. The hemorrhage status was classified as: no hemorrhage, intraventricular hemorrhage, and intracerebral hemorrhage. The PT_APTT was categorized into normal and abnormal levels. The outcome was defined as the survival or death of the neonates.

### 2.2. Setting and Study Design

This study utilized a Bayesian spatial survival modeling approach using secondary data from NFS cases. Based on the geographical location of residence of neonates who were admitted to the NICU at the Kilpauk Medical College and Hospital, Chennai, Tamil Nadu, India, their locations were grouped into six blocks within the Tiruvallur district. Although the Tiruvallur district comprises a total of 14 blocks, the neonates were admitted from only 6 specific blocks: Tiruvallur, Poonnamalle, Villivakkam, Minjur, Puzhal, and Sholavaram.

### 2.3. Ethics Approval

The original study was approved by the Institutional Ethical Committee of the Government Kilpauk Medical College and Hospital (IEC No. 02A-2017.14/11/2017).

### 2.4. Statistical Analysis

The proportional hazard (PH) frailty model was applied to the data on the neonates with fungal sepsis. The event of interest was defined as the death of neonates with fungal sepsis who were admitted to the NICU at the hospital. In this analysis, right censoring was used, and the time was defined as the difference between the time of admission to the NICU, and either the time when the event of interest occurred or the point of discharge. The frailty model incorporated a random effect to capture unobserved factors influencing the hazard, allowing for individual or group-level variability [26].

Parametric frailty models, including the Log-logistic PH [27,28,29], Log-normal PH [27,30,31], and Weibull PH [27,31,32,33] models, were applied to fit the data by incorporating the MCMC iteration, and the results were then compared [34]. In a Bayesian framework, estimating model parameters was the primary objective of the MCMC methods, such as Metropolis–Hastings [35] and Gibbs sampling [36] algorithms, which generated samples from the appropriate marginal posterior distributions. The MCMC sampler explored a parameter’s space based on its prior knowledge and the likelihood of the observed values [37]. Plotting the values in the simulated chains against the iteration, known as the MCMC trace plot, was used as a diagnostic tool to check for the model convergence. A high degree of chain mixing suggested that the MCMC had converged. In this study, a non-informative prior was used in the Bayesian analysis [38].

The fitted survival curve was employed to gain a better insight into the geographic pattern [39,40]. Cox–Snell residual plots [15,40,41] were used to assess the models’ fitness and to compare the fitted models. It was assumed that each region had at least one neighbor; hence, the proportionality constant for the improper density, representing the non-spatial data, was considered under an independent Gaussian prior [14,40]. Three measures—Log pseudo marginal likelihood (LPML), the Deviance Information Criterion (DIC), and the Watanabe Akaike Information Criterion (WAIC) [15,38]—were used to compare the best fit model, with the model having the lowest LPML, DIC, and WAIC being selected as the best Bayesian spatial PH frailty model.

The prior for the regression coefficient of the PH model followed a normal distribution with a mean of ‘0’ and a large variance of ‘1000’. The prior for the frailty term (or random effect) followed a Gaussian distribution with a mean of ‘0’ and the default variance used in the R software. The spatial prior was an independent and identically distributed random effect that followed a normal distribution with a mean of ‘0’ and a standard deviation of ‘σ’. It accounted for heterogeneity after adjusting for subject-specific covariates and included a random effect within each group [15,38]. Statistical analyses were performed using the R version 4.1.3 software. The spatial map was created using the Quantum Geographic Information System (QGIS) version 3.26 software.

## 3. Results

### 3.1. Demographic and Clinical Details for the NFS Data

The descriptive analysis of associated parameters, such as the gestational age in weeks, the neonates’ birth weight in kilo gram (kg), and platelet counts in microliters (10^3^/μL) with respect to the survival and death of the neonates (Table 1). The mean birth weight was 2.08 kg. The platelets counts of the neonates ranged from 2 to 252 (10^3^/μL). The average values of hemorrhages and PT_APTT levels for neonates who died were 0.86 and 0.96, respectively, which were higher than those for neonates who survived. In contrast, the average platelet count for the neonates who died was 47 (10^3^/μL), which was lower compared to the platelet count of the neonates who survived (56.4 (10^3^/μL)).

### 3.2. Posterior Estimates of Spatial Frailty in the Three PH Models

The Bayesian spatial frailty estimates for the three models were obtained using 1000 iterations after discarding first 1000 burin in iterations. The posterior estimates for the three PH models, along with the spatial frailty hazard ratios and their 95% credible intervals (CI), are presented in Table 2. We identified the risk factor of death for neonates for each model separately. In the table, a 95% credible interval of any variables which did not include zero was considered as a significant factor (*p* < 0.05). We considered only the significant variables for further model comparison. NFS cases that had abnormal PT_APTT levels faced a substantially higher risk of death compared to those with normal PT_APTT levels across all three models (Log-logistic PH model: HR = 22.12, 95% CI: 5.40–208.08; Log-normal PH model: HR = 20.87, 95% CI: 5.29–123.23; Weibull PH model: HR = 18.49, 95% CI: 5.60–93.41). Overall, the risk of death was more than 18 times higher in neonates with abnormal PT_APTT levels.

Additionally, fungal sepsis in neonates with the presence of hemorrhages had an increased risk of death compared to those without hemorrhages in the Log-normal and Weibull models (Log-normal PH model: HR = 1.65, 95% CI: 1.05–2.75; Weibull PH model: HR = 1.75, 95% CI: 1.07–3.12) while the Log-logistic PH model did not show a significant association. Overall, the risk of death was approximately 1.75 times higher in neonates with hemorrhages.

Abnormal PT_APTT levels are a much stronger predictor of mortality (HR > 20) than hemorrhages (HR ~1.65–1.75).

**Figure 1:**
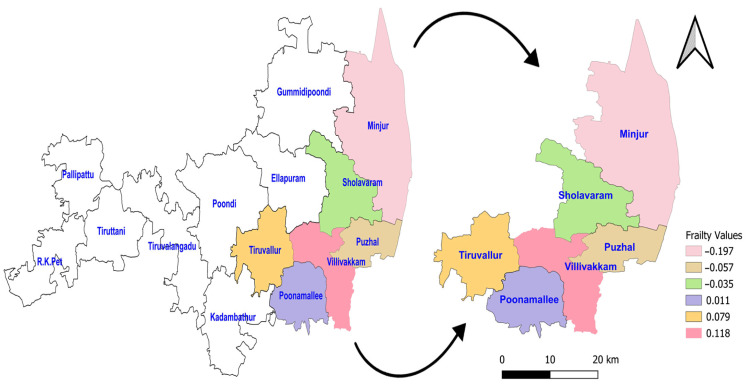
Posterior frailties for the NFS data obtained from the Weibull model for six blocks of the Tiruvallur district. The arrow represents the 6 blocks Tiruvallur district from the 14 blocks. Another symbol represents the directions (north arrow).

**Figure 2:**
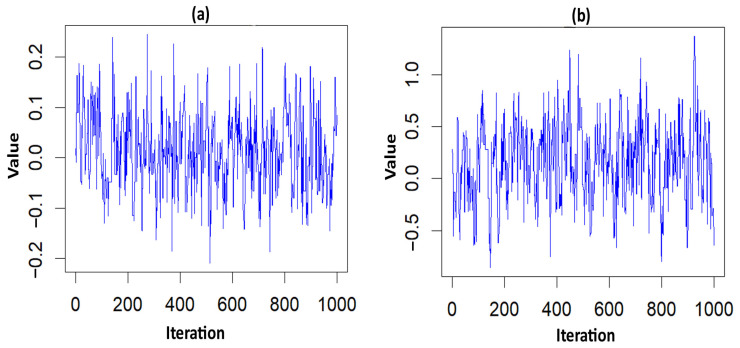
Trace plots of the significant parameter for the NFS data. (**a**) hemorrhage; (**b**) PT_APTT.

**Figure 3:**
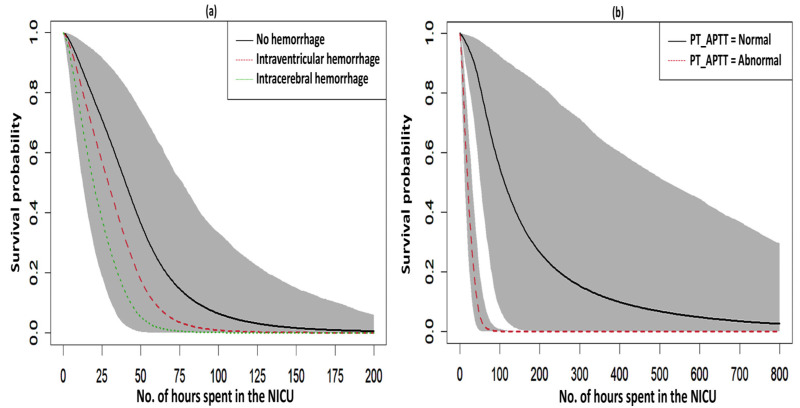
Survival curves with a 95% credible interval for the significant factors in NFS. (**a**) hemorrhage and (**b**) PT_APTT.

The acceptance rate of the Adaptive Metropolis–Hastings Algorithm for the three PH models—such as Log-logistic, Log-normal, and Weibull—were 0.17, 0.20, and 0.19, respectively, which is around 0.2 and is considered a practical acceptance rate for the sampling process. The results of the fit indices (LPML, DIC, and WAIC) are presented in Table 3. Based on the fit indices of the three models, they were identified as good fits for this data. Among them, the Weibull model had the lowest fit indices value (LPML = 220.68, DIC = 439.64 and WAIC = 440.76) when compared to the other two models, so it was further explored. The traces plots, spatial frailty map, and survival curves for this model are presented in Figure 1, Figure 2 and Figure 3. Figure 1 represents the average values of the posterior sampling frailties. The positive posterior frailty values indicate an increase in the risk of disease outbreak, while negative values indicate a decrease in the risk of disease outbreak.

The higher rates of mortality for NFS cases occurred in the hotspot regions of Villivakkam (0.118), Tiruvallur (0.079), and Ponnamallee (0.011) among the six blocks within the study area. The spatial frailty model effectively explained the differences in infant mortality from fungal sepsis among the blocks. The trace plot of the parameters from the Weibull PH spatial frailty model displayed a narrow horizontal band, indicating that the parameters had converged, as shown in Figure 2.

**Figure 4:**
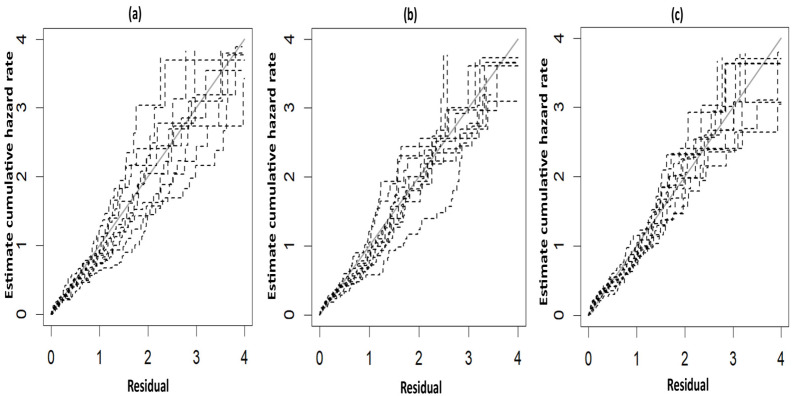
Cox–Snell plots for the three PH frailty models in NFS. (**a**) Log-logistic; (**b**) Log-normal; (**c**) Weibull.

The fitted survival curves with 95% CIs are depicted for a GA_weeks of 30 weeks, a B_Weight of 1 kg, a platelet count of 25 (10^3^/μL), and abnormal PT_APTT levels across different types of hemorrhage (Figure 3a). The plot indicates that survival probability is higher at earlier time points but decreases as time progresses. Additionally, it demonstrates that survival probability is lower for intracerebral hemorrhages compared to other types of hemorrhage. Another set of fitted survival curves with 95% CI is shown for a GA_weeks of 30 weeks, a B_Weight of 1 kg, a platelet count of 25 (10^3^/μL), and an intracerebral hemorrhage at varying PT_APTT levels (Figure 3b). The figure reveals that survival probability is initially higher but declines over time. It also shows that when PT_APTT is abnormal, survival probability approaches zero after 60 h. In contrast, when PT_APTT is normal, survival probability remains significantly higher compared to when PT_APTT is abnormal. The Cox–Snell residual plots for the three PH models, which were used to assess the reliability of these models, showed nearly straight hazard plots with a slope of one, suggesting a good model fit, as visualized in Figure 4.

## 4. Discussion

This research article has demonstrated Bayesian spatial frailty models based on the geographic locations of neonates admitted to the NICU at Kilpauk Medical College and Hospital, Chennai, Tamil Nadu, India. To our knowledge, there has been no previous studies on Bayesian spatial frailty models in the survival of neonates with fungal sepsis. This study used a novel approach to model the spatial dependence of survival times of NFS cases. We assessed the posterior estimates of the parameters for the three frailty PH models—Log-logistic, Log-normal, and Weibull—and validated these models using fit indices for model selection. We also identified clinical factors that impacted geographical disparities in neonatal mortality. Although all the models performed well, the Weibull PH model had the lowest fit indices, representing spatial frailty and mortality patterns in NFS. Our key findings revealed spatial heterogeneity, which had a major impact on the severity and mortality of NFS cases, with the presence of hemorrhage and abnormal PT_APTT levels. Our study has highlighted the elevated neonatal mortality rates in the hotspot blocks of Tiruvallur, Villivakkam, and Ponnamallee in the Tiruvallur district.

A similar finding regarding the better performance of the Weibull model was documented in a study on Bayesian spatial survival models for dengue hospitalization at Wahidin Hospital in Makassar, Indonesia [10]. The results of the current study are aligned with prior studies that revealed the advantages of spatial survival models in various contexts, including dengue patient survival, mortality risks in under-5s, and COVID-19 recovery times [14,42,43]. Another study on pediatric leukemia from Southern Iran reported that the Log-normal and Weibull models were found to be superior to the Cox regression model [44]. A study from Mexico on a spatial survival model for COVID-19 highlighted that the spatial model outperformed the Cox model in capturing variations in survival across the Mexican states. Regional clusters showed distinct mortality risks, demonstrating the necessity of geographical locations analysis for accurate risk assessments and resource allocations [15]. Additionally, another study using a Bayesian spatial model concluded that the prevalence of TB/HIV co-infection varied spatially [45].

In this study, the fatalities of NFS cases were found to be 62.5%. Neonatal mortality among preterm newborns in NICUs in Pakistan was twice as high as in Indian NICUs, according to a prospective study carried out in NICUs at three hospitals in Davangere, India, and a public hospital in Karachi, Pakistan. This occurred because NICUs in Pakistan had fewer diagnostic tests, shorter NICU stays, and fewer resources [46].

The observed geographical dependency in survival outcomes highlighted the importance of considering a neonate’s birthplace when developing targeted interventions, such as supporting pregnant women and closely monitoring high-risk pregnancies to address neonatal mortality effectively. Spatial survival models could have aided obstetricians and healthcare providers in identifying high-risk blocks within the district that required additional resources and focused management strategies, thereby reducing preterm births, lowering infant mortality, and preventing complications associated with fungal sepsis. NFS is strongly linked to preterm birth, and infant death rates may be considerably reduced by recognizing and controlling risk factors, such as PT_APTT levels and hemorrhages. Postnatal interventions are also crucial in mitigating the risk of neonatal death.

There were a few limitations in this study. The absence of important clinical factors, such as the methods of delivery, the hematological characteristics, and the biochemical characteristics of the pregnant women, may have limited the model’s accuracy. Furthermore, there may be bias in misclassification due to the dependence on self-reported data for comorbidities. Socio-demographic factors, such as maternal age, education, income, pregnancy intervals, and access to essential services, were also absent, further constraining our analysis. Maternal infections in healthcare settings remained a critical contributor to neonatal mortality and morbidity [47]. In addition, there may be over 80 NFS cases in these zones, and there is the possibility of admission to nearby or other hospitals, which presents another limitation for this study.

The limitations on the spatial frailty model are as follows: non-informative priors were used in this study and assumptions about priors have an impact on the outcome. The frailty estimates depend on the spatial structure and the inclusion of covariates. The findings of the study may vary in different study setups and geographical regions.

Future research should aim to address these limitations by incorporating socioeconomic indicators, levels of urbanization, poverty data, climatic factors, and additional clinical and laboratory parameters. Expanding the study to include data from more districts and states will provide a broader understanding of NFS’s survival determinants. To enhance our understanding of these determinants and improve strategies for comprehensively evaluating and treating NFS, data from other hospitals in Tamil Nadu, India, should be studied.

## 5. Conclusions

In this study, we assessed the survival times associated with NFS cases across six blocks in the Tiruvallur district of Tamil Nadu, India, taking into account the geographic distribution of NFS. Our results showed that all three frailty PH Models—Log-logistic, Log-normal, and Weibull—fitted the NFS data well, although the Weibull model performed slightly better than the other two. The substantial variations in survival times by geographic location further emphasized the importance of spatial determinants in NFS survival.

We found that NFS cases had a higher risk of mortality if they exhibited abnormal PT_APTT levels or the presence of hemorrhages. These findings underscore the critical need for the early identification and close monitoring of newborns with these risk factors in NICUs. Prompt intervention, combined with targeted care strategies, may significantly improve survival rates. To reduce mortality in NFS cases, future initiatives should incorporate these findings into NICU protocols, with an increased focus on risk stratification, early diagnostic screening, and evidence-based interventions.

## Figures and Tables

**Table 1:** Descriptive statistics of demographic and clinical data for neonates with fungal sepsis.

	Parameters	Min	Q_1_	Median	Mean	Q_3_	Max	SD
Survived (N = 30)	GA_weeks	28	32	36.5	35.47	38	42	3.69
	B_WEIGHT	1	1.49	2.25	2.11	2.46	3.3	0.62
	Platelet	2	14	31	56.4	62.75	252	67.31
	Hemorrhage	0	0	0	0.27	0	2	0.69
	PT_APTT	0	0	0	0.1	0	1	0.31
Died (N = 50)	GA_weeks	26	31	35.5	34.5	38	40	4.24
	B_WEIGHT	0.76	1.47	2.1	2.06	2.73	3.96	0.77
	Platelet	3	8.75	24.5	47	64	232	50.98
	Hemorrhage	0	0	1	0.86	2	2	0.9
	PT_APTT	0	1	1	0.96	1	1	0.2
Total (N = 80)	GA_weeks	26	32	36	34.86	38	42	4.05
	B_WEIGHT	0.76	1.49	2.2	2.08	2.72	3.96	0.72
	Platelet	2	11	25.5	50.53	61.75	252	57.41
	Hemorrhage	0	0	0	0.64	2	2	0.88
	PT_APTT	0	0	1	0.64	1	1	0.48

Min: minimum; Max: maximum; SD: Standard deviation; Q_1_: lower quartile; Q_3_: upper quartile; GA_weeks: Gestational age (in weeks); B_WEIGHT: Birth weight (in kg); Platelet (in 10^3^/µL); PT_APTT: Levels of activated thromboplastin level.

**Table 2:** Posterior estimates of parameters of the three frailty models for neonates with fungal sepsis.

Model	Parameters	Mean	Hazard Ratio (HR)	Median	SD	95% CI (Mean)	
						Lower	Upper
Log-logistic	GA_Weeks	0.02	1.02	0.02	0.07	−0.12	0.16
	B_WEIGHT	0.12	1.12	0.13	0.36	−0.59	0.81
	Platelet	0	1	0	0	−0.01	0.01
	Hemorrhage	0.56	1.75	0.58	0.3	−0.02	1.16
	PT_APTT **	3.1	22.12	3.03	0.84	1.69	5.34
Log-normal	GA_Weeks	0.03	1.03	0.03	0.07	−0.1	0.16
	B_WEIGHT	0.06	1.06	0.07	0.34	−0.64	0.67
	Platelet	0	1	0	0	−0.01	0.01
	Hemorrhage **	0.5	1.65	0.5	0.26	0.05	1.01
	PT_APTT **	3.04	20.87	2.98	0.79	1.67	4.81
Weibull	GA_Weeks	0.01	1.01	0.01	0.07	−0.14	0.15
	B_WEIGHT	0.18	1.2	0.2	0.33	−0.48	0.78
	Platelet	0	1	0	0	−0.01	0.01
	Hemorrhage **	0.56	1.75	0.56	0.27	0.07	1.14
	PT_APTT **	2.92	18.49	2.87	0.74	1.72	4.54

** *p* < 0.05.

**Table 3:** Fit indices of three frailty PH models for model selection.

Model	LPML	DIC	WAIC
Log-logistic PH Model	221.65	441.33	442.83
Log-normal PH Model	221.89	442.05	443.51
Weibull PH Model	220.68	439.64	440.76

## Data Availability

The data will be made available upon receiving written request and approval granted by the study authority.

## Abbreviations

The following abbreviations are used in this manuscript:

NFS	Neonatal Fungal Sepsis
NS	Neonatal Sepsis
PH	Proportional Hazard
NFHS-5	National Family Health Survey—5
NNMR	Neonatal Mortality Rate
NICU	Neonatal Intensive Care Units
PT	Prothrombin Time
aPTT	activated Partial Thromboplastin Clotting Time
PT_APTT	Levels of Activated Thromboplastin Level
ICH	Intracranial Hemorrhage
TB	Tuberculosis
MCMC	Markov chain Monte Carlo
GA_weeks	Gestational Age in Weeks
B_Weight	Neonates Birth Weight
LPML	Log Pseudo Marginal Likelihood
DIC	Deviance Information Criterion
WAIC	Watanabe Akaike Information Criterion
QGIS	Quantum Geographic Information System
CI	Credible Intervals
HR	Hazard Ratio
LOS	Late-Onset Sepsis
